# A Single-Layer PDMS Chamber for On-Chip Bacteria Culture

**DOI:** 10.3390/mi11040395

**Published:** 2020-04-10

**Authors:** Pablo Morales Navarrete, Jie Yuan

**Affiliations:** Department of Electronic and Computer Engineering, Hong Kong University of Science and Technology, Kowloon, Hong Kong

**Keywords:** single layer valve, PDMS microfluidics, whole-cell biosensor

## Abstract

On-chip cell culture devices have been actively developed for both mammalian cells and bacteria. Most designs are based on PDMS multi-layer microfluidic valves, which require complicated fabrication and operation. In this work, single-layer PDMS microfluidic valves are introduced in the design of an on-chip culture chamber for *E. coli* bacteria. To enable the constant flow of culturing medium, we have developed a (semi-)always-closed single-layer microfluidic valve. As a result, the growth chamber can culture bacteria over long duration. The device is applied for the whole-cell detection of heavy metal ions with genetically modified *E. coli*. The platform is tested with culturing period of 3 h. It is found to achieve a limit-of-detection (LoD) of 44.8 ppb for Cadmium ions.

## 1. Introduction

Microfluidic on-chip cell culture has been actively investigated in the past two decades. The idea of having an automated and customized cell culture environment has been actively pursued and would bring many advantages to researchers that work with live cell samples [1], such as the low reagent consumption, automation and the customizable cellular environments [2]. 

Hung et al. presented the first cell culture microfluidic device aimed at providing a high throughput for cell-based experiments [3]. This device was able to culture Human carcinoma (HeLa) cells over several days. Additionally, with the introduction of a chemical gradient generator, Hung et al. were able to create an array of different conditions in which HeLa cells were tested [3]. This device, alongside the work by Leclerc et al., enabled a better gas interchange due to the inherent high gas permeability of the PDMS material they used in their devices. This helped overcome the poor oxygen distribution to the cells of previous cell-culture work. [3,4,5]

Early on-chip culture devices had all their chambers interconnected which meant that the individual manipulation of each chamber was not possible. The interconnection of culture chambers produces cross contamination with other chambers altering the results of the cellular culture [6]. To overcome this problem, Gómez-Sjöberg et al. developed a fully automated microfluidic cell culture system where each individual cell culture chamber was isolated using PDMS microfluidic push-down valves [7]. Overtime, this architecture was adopted by many and has set the grounds for works such as organ-on-a-chip [8], automated single cells studies [9], 3D culture studies [10], long-term tumor response to drugs [11], among others.

The first bacteria on-chip culture devices developed would cause cellular stress and trigger bacterial biofilm formation, causing problems in the bioreactor and interfering with the results of the bacterial cell culture [12]. To address this problem, Balagaddé et al. developed a complex microfluidic chip that allowed for continuous medium flow, reducing the risk of biofilm formation [13]. Continuous advances in automated miniaturized bacterial culture systems have brought us to the current stage of maturity of bacterial bioreactors. Currently, these miniaturized culture systems are being used in many diverse applications such as microbial antibiotic resistance [14], high-throughput analysis [15,16], among others. Nonetheless, existing on-chip culture bacteria chambers are normally designed in multiple layers, making the device hard to manufacture [7].

Most existing devices trade functionality for fabrication complexity, increasing the number of peripheral devices needed to operate the device. The main reason behind this complexity is the use of push-up or push-down microfluidic valves. These valves utilize compressed air to selectively manipulate fluid flow [17]. Microfluidic valves are fabricated using multiple layers of PDMS which must be correctly fabricated and aligned to avoid any scaling or misalignment issues. A misalignment between two or more layers would completely render the device useless [17]. A microfluidic on-chip culture device that could continually flow medium through the growth chamber while keeping the fabrication method simple and reliable would allow for bacterial bioreactors to be used in settings with an absence of specialized equipment. Such a device would permit for the manipulation of bacterial cells while minimizing the complexity of the operation and fabrication, bringing down the time, skill and cost per device.

Single-layer valves are a technology that enables the manipulation of fluids by actuating pneumatic channels with pressurized air. Traditional valves use compressed air or N_2_ to deform a PDMS membrane to shut a microfluidic channel. Alternatively, single-layer valves control channels by laterally deforming the PDMS membrane [18,19,20]. These single-layer valves are always open valves since they require an external actuation to close off the channel. Continuous pressurization is required when the channel is kept close. However, in some applications this is required most of the time. A good example is a reaction chamber. In this case, the chamber must be regularly isolated to enable the reaction to take place in the chamber. If always-open valves are employed, complete sealing must be achieved to avoid leakage. As such, continuous pressurization is needed, which is not always ideal and could incur in valve failure after multiple cycles [21]. Additionally, these valves are normally actuated by voluminous pneumatic systems comprised of canisters of compressed air, rendering the device immobile. Always-closed valves can be designed as an alternative, simplifying the chip’s valve actuation [21,22]. It would reduce the continuous actuation of the valve, prolonging the feasibility of the device and reducing the fatigue of the PDMS. To our knowledge, no single-layer always-closed valve design exists.

In this manuscript, we introduce our design of a PDMS chamber for culturing bacteria using a single-layer valve. By developing a new single-layer semi-always-on valve, the on-chip culture chamber can be fabricated in one layer of PDMS, reducing the fabrication complexity. This chip provides an advancement in comparison with existing cell culture solutions because it offers a simple and reliable design to enable fluid handling and cell culture automatization. Additionally, the incorporation of a low cost, and accessible actuation method allows the use of these type of microfluidic chips in environments with low resources. The culture chamber is used as a whole-cell biosensor to detect heavy metal ions, such as Cadmium [23,24,25]. With this new culture chamber, the genetically modified *E. coli* can be cultured continuously for 3 h in the water sample. It can detect Cadmium ions with an LOD of 44.8 ppb [25].

## 2. Design

### Microfluidic Device Design

**Figure 1:**
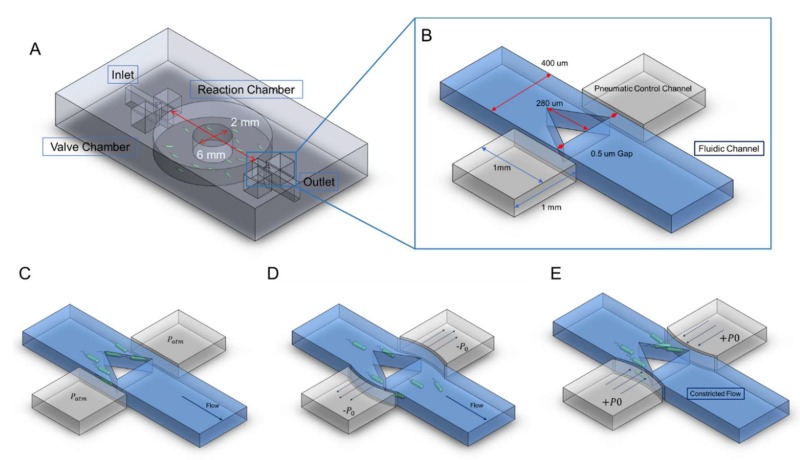
Schematic of the microfluidic device and working principle of the semi-always-closed valve. (**A**) The microfluidic device consists of a large central chamber, a fluidic channel and several pneumatic control channels. (**B**) Close up of the single layer semi-always closed valve. (**C**) In an unaltered state, the opening to the sides of the microfluidic structural element will allow the medium to pass while blocking the pass of bacteria. (**D**) When we subject the pneumatic control channels to negative pressure, the membrane deforms allowing the bacteria to pass freely. (**E**) When we subject the control channels to positive pressure, the membrane will deform cutting off the flow of the medium.

Our device is designed to maintain the *E. coli* cells viable for 3 h with running medium. Consequently, we designed a large reservoir where sensing bacteria would grow with the sample medium (see Figure 1A). The central reservoir has an approximate volume of 1 μL connected by two channels measuring 400 μm in width, ~30 μm in height and several mm in length. The inlet and outlet to the central chamber have the side pneumatic channels selectively controlling the flow of the medium or the bacterial sample (see Figure 1A).

As shown in Figure 1B, the microfluidic valve consists of a central triangular PDMS structure that serves as the structural element closing off the channel. We designed the triangular structural element in such a way to redirect the flow to the sides of the channel and thus maintain the flow resistance to a minimum. With other geometries, the flow would have been more heavily disturbed, creating unnecessary flow disruptions. The central element has a 0.5 μm side opening at its edges (see Figure 1B) to allow the perfusion of medium, while simultaneously blocking off the bacteria. Adjacent to the valve element, there are two pneumatic chambers separated by a membrane with 20 μm thickness. These pneumatic chambers will be inflated or deflated with compressed air to achieve varying pressures. In turn, the flexible PDMS side walls in proximity to the valve element will deflect, opening the gap of the microfluidic channel to allow *E. coli* cells to pass or closing the gap to completely seal off the channels connected to the culture reservoir (see Figure 1C–E).

With simple syringe pumps, the pneumatic channels can be accordingly depressurized at specific values to control the deflection of the side PDMS membrane. The ability to control the deflection of the thin membrane enables us to selectively control the beads or cells to pass as a controllable size filter. This can find usage in a wide range of applications [26]. 

## 3. Materials and Methods

### 3.1. Microfabrication

**Figure 2:**
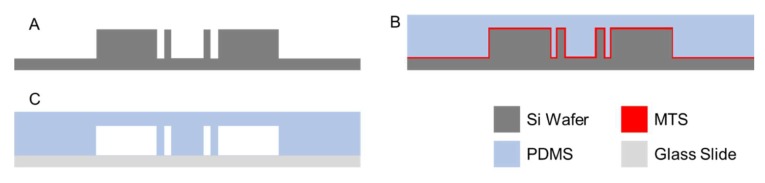
Microfabrication process flow for the fabrication of the single-layer microfluidic device. (**A**) DRIE etch of Si wafer. (**B**) PDMS casting over Si stamp. (**C**) Microfluidic device assembly on glass slide.

The semi-always-closed single-layer microfluidic valve was built using soft-lithography on a single silicon mold [27]. The mold was fabricated by etching a silicon wafer using DRIE, achieving a depth of ~30 μm and up to 0.5 μm in the narrowest section of our microfluidic channel (see Figure 1). Firstly, a silicon wafer was cleaned, and spin coated with HPR 504 photoresist (PR) in preparation for photolithography. Then, the wafer was exposed to UV light using the Karl Suss MA6 Desktop Aligner (SÜSS MicroTec, Garching, Germany). Once the photoresist mask was developed, we etched for 30 min using DRIE Bosch Process achieving a depth of ~30 μm (see Figure 2A). After the silicon wafer was etched, the PR was stripped, and the silicon wafer was cleaned using H_2_SO_4_. Many previous works involving microfluidics use photoresists such as SU-8 as the master mold. SU-8 is widely used due to the high aspect ratio this material can achieve. Nonetheless, SU-8 molds degrade rapidly under successive uses. On the other hand, etched Si molds are very robust and can withstand multiple rounds of PDMS casting allowing the user to rapidly prototype PDMS microchannels. Hence, DRIE etched silicon is used as the master mold in our work 

Once the silicon stamp was finalized, the silicon wafer was treated with methyltrichlorosilane (MTS) for a few minutes to allow an easy pealing of the PDMS after curing. The surface modification was performed by depositing several milliliters of methyltrichlorosilane on a petri dish in an enclosed process chamber containing the silicon wafer. The wafer was left in the process chamber for several minutes to ensure a good surface treatment. 

A mixture of 10:1 PDMS to curing agent was poured onto our silicon mold and left to cure for 2 h at 80 °C. Once the PDMS was fully cured, the PDMS layer is peeled off. The device is cut, punched for inlet/outlet holes and bonded onto a glass slide using a plasma cleaner. For our biosensor experiments, we bonded several devices together on a single glass slide to create an array of chambers to test multiple sample solutions with varying concentrations of heavy metal particles simultaneously. 

### 3.2. Growth and Preparation of E. coli

**Figure 3:**
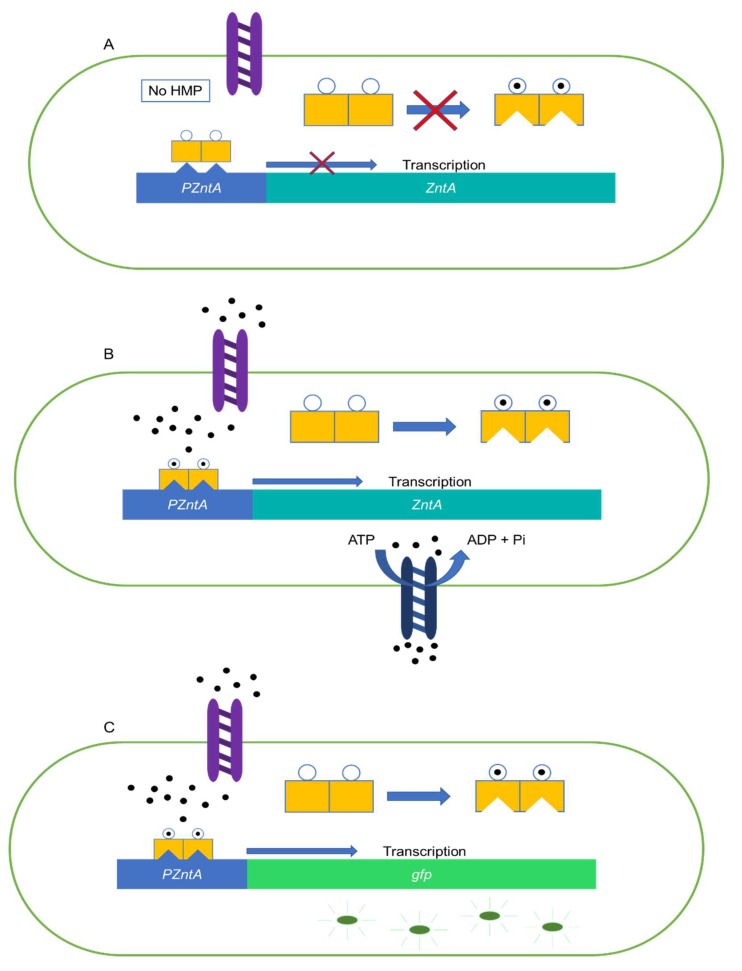
Znt regulation mechanism. (**A**) Znt regulation mechanism in absence of heavy metal ions. (**B**) Znt mechanism in presence of heavy metal ions. (**C**) Genetically modified bacteria synthesing gfp under the presence of heavy metal particles.

The sensing agent for our heavy-metal-ion sensor is an inducible *E. coli* DH5αPRO from Professor Timothy K. Lu’s group in MIT. The cells were transformed with recombinant plasmids carrying a *pzntA-gfp* gene fusion construct. The *Znt* operon is a group of genes that is present in several types of bacteria and is responsible for the efflux mechanism of heavy metal ions from the cellular cytoplasm. This mechanism acts as a selective removal system capable of lowering the concentrations of heavy metals such as Zn^2+^, Cd^2+^ and Pb^2+^ present in the cell by actively flowing these heavy metals from the cellular cytoplasm by means of transport proteins (see Figure 3). One of the proteins involved in the transport of these heavy metals is *zntA*, which is responsible for the efflux of Zinc in *E. coli*. In the presence of heavy metal ions, the transcription factor of *ZntA* (*ZntR*) is able to promote the transcription of the *zntA* transport protein, which is responsible for the removal of heavy metal ions from the cytoplasm (see Figure 3B). On the other hand, when there is an absence of heavy metal ions in the cytoplasm, the transcription regulator cannot initiate the transcription of the ZntA gene and thus does not synthesize the desired protein. 

During the genetic modification, a *gfp* gene was fused downstream of the *pzntA* gene. Therefore, once the modified bacteria take into its cytoplasm the heavy metal ions, the *ZntR* will trigger the transcription of the *gfp* gene, causing the bacteria to synthesize GFP which will generate a detectable light signal (see Figure 3C).

For our biosensing experiments, we cultured *E. coli* in LB Broth (Sigma-Aldrich) and incubated overnight at 37 °C in a shaker-incubator. On the following day, we centrifuged and resuspended the cells in fresh medium to an OD600 ≈ 0.7. Once the sample was ready, we filled a syringe with the cells and connected it to the microfluidic device. 

### 3.3. Experimental Setup

Once the device was fabricated and assembled, we connected 25 mL syringes to Tygon (Sigma-Aldrich) tubing to control the volume of air pumped in and out the control channels. A syringe pump is mounted to accurately displace the syringe plunger. Additionally, we connected the inlet of the microfluidic device with Tygon tubing to a supplementary syringe pump to control the flow rate of our medium and cell sample.

With syringe pumps, the pressure in the pneumatic control channels changes according to following equations.

$$P_{final}=\frac{P_{0}\cdot V_{0}}{V_{final}}$$

$$∆P=P_{final}-P_{0}=P_{0}\cdot \frac{1}{\frac{V_{0}}{∆V}-1}$$

Where $P_{0}$ is the atmospheric pressure, which is also the pressure in the main fluidic channel without fluid. ∆P is the pressure difference across the membrane. By controlling the ratio of the air volume change in the side channel, this pressure difference can be precisely set. In our experiments, the syringe volume varies from 0 mL to 25 mL. Hence, the pressure difference varies from 0 to ~97 kPa. With the syringe pumps, the single-layer valves in this device can be accurately controlled without using more complicated electropneumatic systems such as solenoid valves and compressed gas canisters. 

**Figure 4:**
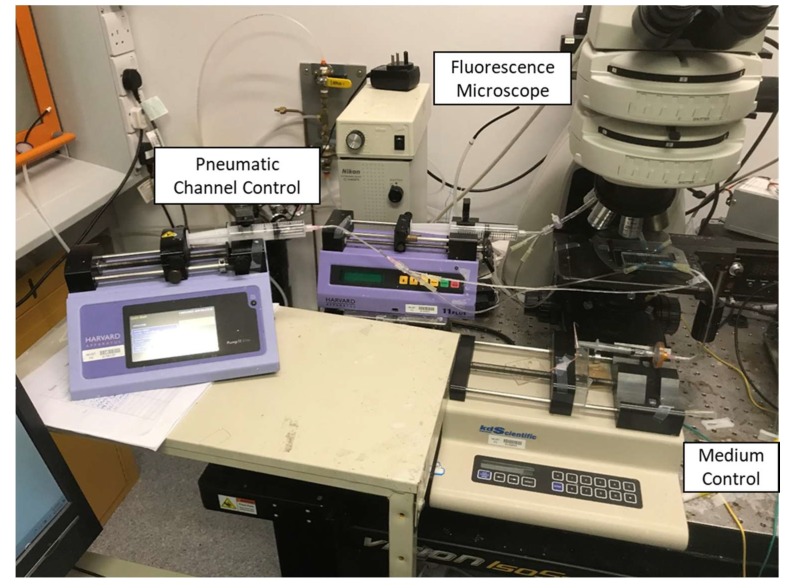
Experimental setup containing the syringe pumps to actuate the device.

For our Cadmium sensing experiment, we seeded the reaction chambers with the recombinant *E. coli* by opening the valve of the inlet. Once the growth chamber was successfully seeded, the valve was kept in its rest state and we perfused medium into the chamber alongside a sample of Cadmium heavy metal ions. We kept the device at room temperature and measured the resulting fluorescence with a fluorescence microscope 3 h later (Nikon Eclipse Ni-U, Nikon, Tokyo, Japan). Alternatively, to quantify the temporal evolution of the biosensor, we measured the fluorescence over a period 3 h in 15-min intervals. The full experimental setup containing the syringe pumps to control the actuation of the microfluidic device can be observed in Figure 4.

## 4. Results

### 4.1. Valve Performance

**Figure 5:**
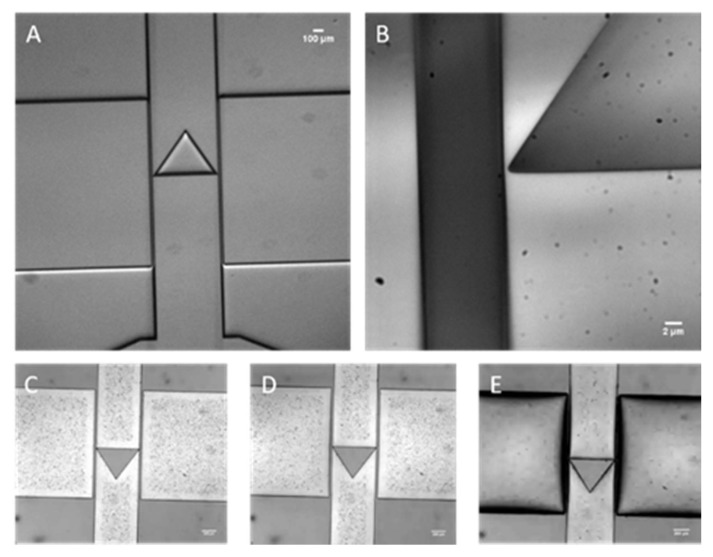
Brightfield images of silicon micromachined mold (**A**,**B**) and PDMS single layer valve under different actuation pressures (**C**,**D**). (**A**) Brightfield image of micromachined silicon stamp for soft lithography. (**B**) 100x magnification of the corner of our structural element. As it can be observed the side opening measures ~0.5 μm. (**C**) Brightfield image of our microfluidic valve in the rest state. (**D**) Brightfield image of our microfluidic valve actuated at a negative pressure of 0.5 atm. (**E**) Brightfield image of our microfluidic valve actuated with a positive pressure of 1 atm.

Microscope images of the silicon mold and the fabricated PDMS valve device are shown in Figure 5A–E. The 0.5μm gap is visible along with the 20 μm thick membrane. Figure 5C shows the membrane at the rest pressure. Figure 5D,E show microscope images of the membrane with a negative and positive actuation pressure respectively. The membrane experiences its greatest deformation at the center in the vertical direction. Therefore, it exhibits a bulging shape with a negative pressure. The microscope image shows clear thickening of the membrane in Figure 5E when the deformation is large. 

The functionality of the valve can be visualized with the help of fluorescent beads. In these experiments, polystyrene beads of different sizes were added to the fluidic channel. Sizes of choice were 0.2 μm, 2.5 μm, 5 μm and 10 μm. We can observe that 0.2 μm beads flow without any difficulty through the 0.5 μm gap (see Figure 5A) with the valve at rest. However, with larger beads such as 2.5 μm and 5 μm the beads will get trapped at the sides of the fluidic channel (Figure 5C) with the valve at rest. It is only when the negative pressure in the side channel increases to deflect the membrane wide enough so that these beads will be able to flow (Figure 5D). For beads measuring 10 μm, we can observe a flow in the fluid, which is evidenced by the blurred image of a moving bead in the circled area (see Figure 5D). But the beads remain completely blocked even with the negative pressure added to the side channels. This is because the size of the beads has already exceeded the maximum possible deflection of PDMS side wall in our design.

Alternatively, other forms of actuation with the use of rubber bulbs could be possible as long as, the actuation of the valve can provide enough pressure difference. In the case of rubber bulbs, enough pressure can be generated. However, rubber bulbs do not have an adequate tubing interface such as Luer lock syringes, resulting in a vulnerable sealing. 

### 4.2. Heavy Metal Testing with the Microfluidic Device

**Figure 6:**
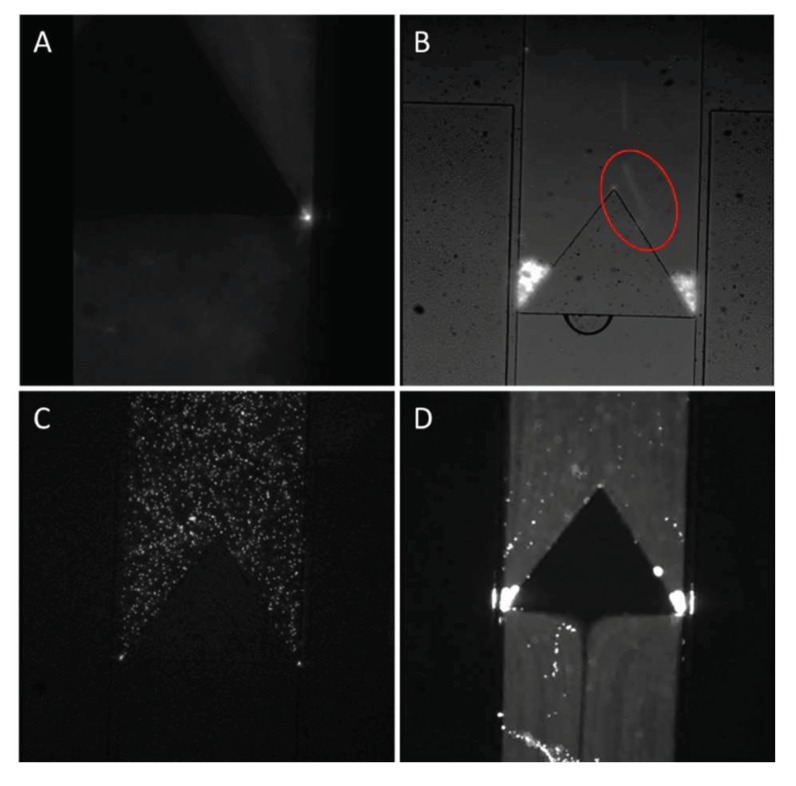
Fluorescent images depicting the flow of fluorescent bead through the semi-always-closed single-layer valve. (**A**) 0.2 μm fluorescent beads diffusing through the valve side opening. (**B**) 10 μm fluorescent beads getting trapped in the microfluidic valve, we can see that the fluid continues to flow due to the shade of a flowing bead (red circle). (**C**) 5 μm fluorescent bead initially getting trapped before actuation of the single layer valve. (**D**) 5 μm fluorescent bead passing through the single layer valve once the side wall was deflected.

*E. coli* were seeded in the growth chamber after opening the valve (see Figure 6A). The cells are cultured over 3 h with running medium through the 0.5 μm gap with the valves at rest. Brightfield and fluorescence microscope images in Figure 6 show that the cells are viable and properly perfused with medium to support growth and synthesis of GFP over the duration of the 3-h inducing period. 

**Figure 7:**
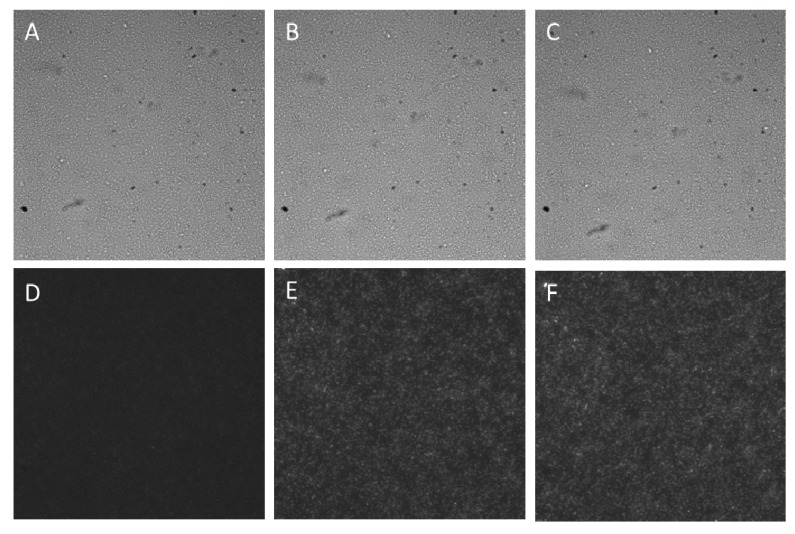
Brightfield and Fluorescence time lapse of cells in the microfluidic growth chamber. (**A**) Brightfield image at t = 0 s. (**B**) Brightfield image at t = 1.5 h. (**C**) Brightfield image at t = 3 h. (**D**) Fluorescence image at t = 0 s. (**E**) Fluorescence image at t = 1.5 h. (**F**) Fluorescence image at t = 3 h.

Once the whole-cell biosensor was seeded in the chamber we induced the cells with cadmium ions. We measured the fluorescence signal over a 3-h period in 15-min intervals. This experiment allowed us to quantify the response time of our biosensor. As recombinant *E. coli* spontaneously generate GFP, a control experiment is applied to measure the fluorescence of *E. coli* cells without adding heavy metal ion solution. Over time, these cells will generate a signal bias that must be considered when analyzing the sample. As it can be observed in Figure 7A, the fluorescence generated by our whole-cell biosensor increases overtime. The results show that 1.5 h after inducing the biosensor, the level of fluorescence is measurable. Additionally, it can also be seen that during a 3h incubation, the levels of GFP do not increase significantly for the control sample. This is because 3 h is not enough to detect a measurable increase in spontaneous GFP. Bacteria divide and populate rapidly over time. From our control experiments visualized on Figure 7A, we can conclude that there is not a notable population increase over a period of 3 h. This can be explained since the sample seeded into the growth chamber has an initial high number of cells (With an OD600 nm of 0.7, there is a bacterial concentration of 5.6 × 10^8^ cells/mL. The 1 µL chamber could contain a total of 560,000 cells.) As the volume is fixed and the cells are tightly packed in our growth chamber, bacterial cells do not expand aggressively like they would do otherwise. Therefore, since the number of cells does not increase rapidly over a 3-h period and the spontaneous GFP is not generated fast enough, the baseline of our sensor does not increase overtime.

High concentrations of cells such as the ones used in this work are needed in whole-cell biosensing since they can reduce the response time, leading to shorter detection and incubation times. In the event of seeding the chambers with a lower concentration of cells, the possibility of detecting low concentrations of target ions in a short incubation time decreases notably [25].

Whole-cell sensors such as the one presented in this work accumulate GFP in their cytoplasm. A notable disadvantage of these biosensors is their inability to remove GFP from their cytoplasm, accumulating over time. To overcome this problem, the culture must be discarded and reseeded to detect additional samples containing heavy metal ions. This is the reason why it is important to have an automatic and simple bacterial microreactor. 

**Figure 8:**
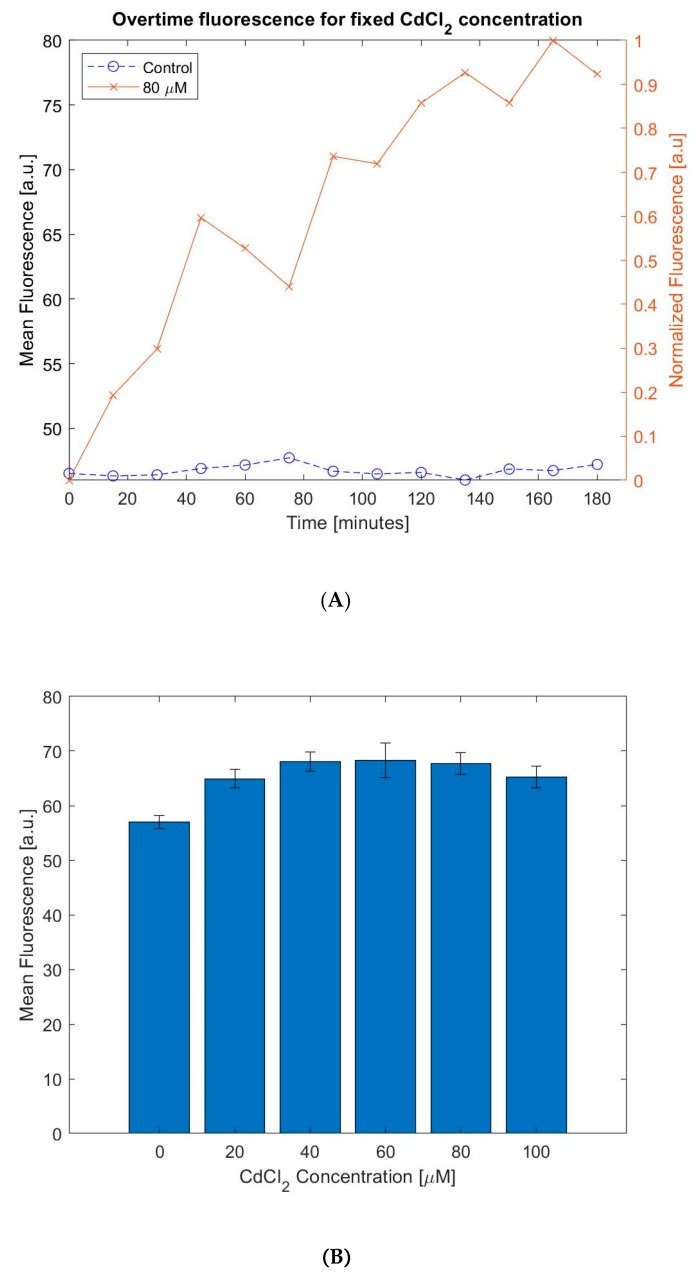
(**A**) Graphical representation of increase in fluorescence for a fixed Cd^2+^ concentration. (**B**) Graph showing the measurement of fluorescence of the proposed device for different concentrations of Cd^2+^.

On the other hand, Figure 7B shows levels of fluorescence with different concentration of cadmium after inducing for 3 h. The values shown on this graph represent the mean and the standard error of the histogram of a fluorescence microscopy image. The experiments on Figure 8B were repeated 4 times on a total of 24 chips. The experiments were run in parallel and fluorescence measurements were taken at three different locations per chip to reduce the variation due to the imaging spot. Our results indicate the fluorescence increases linearly with an increasing cadmium concentration below 40 μM. Beyond 80 μM, our measurements show a clear drop of fluorescence. This phenomenon occurs due to cytotoxicity of cadmium [21]. At higher levels of Cd^2+^ the *E. coli* cells will undergo cellular death due to the high concentration of heavy metal ions in the solution [28]. As it can be observed in Figure 8B, the fluorescence signal peaks at around 60 μM. Nonetheless, between 40 μM to 80 μM, our system shows less sensitivity. This is mainly because our imaging system can only record the fluorescence from a small spot in the reaction chamber for each measurement. Hence, the result is subjected to the miniature cell population variation at the imaging spot in Figure 8B. Using an imaging system with larger field of view, the imaging uniformity could be further improved. Even with this limitation, Figure 8B records similar trends as shown in previous literature [24,25,28]. The limit-of-detection (LOD) of this sensor is about 44.8 ppb for Cd^2+^ [25].

The limits of the biosensor presented in this work were thoroughly explained in previous work [25]. The limit of detection for cadmium ions of this bacterial whole-cell biosensor is 44.8 ppb. Table 1 listed the performance of other whole-cell detection platforms. Our sensor achieved a lower LoD. 

## 5. Conclusions

This paper introduces the design of a single-layer bacterial culturing chamber for whole-cell detection of heavy-metal ions. The device employs a semi-closed valve constructed in a single-layer PDMS. With this valve, the culturing medium keeps flowing through the chamber, which maintains the growth of the sensing bacteria over a long period of time without the need to actuate the valves continuously. The fabrication process and its operation are much more simplified with this single-layer valve. Nonetheless, there are certain drawbacks to the valve presented in this work. The biggest disadvantage of this design is the inability to allow passage to particles larger than ~8 µm. Eukaryotic cells tend to be bigger than bacterial cells. In the current configuration, this device cannot be used on eukaryotic cell assays. This issue can be easily resolved by modifying the design of the valve and changing the spacing between the central structural element and the side walls. 

Genetically modified *E. coli* are seeded in the growth chamber to detect Cadmium ions. Our experiments show that this microfluidic device can culture the bacteria comfortably with a duration of up to at least 3 h. The platform can detect Cadmium with a LOD of about 44.8 ppb. 

## Figures and Tables

**Table 1:** Comparison of Previous Cadmium whole-cell biosensors.

Author	Detector/Reporter	Limit of Detection	Incubation Time	Instrument Used
Our Work [25]	zntA-gfp	44.8 ppb	2–3 h	Custom Detection Platform
Gireesh et al. [24]	zntR-zntA(*E. coli*)-gfp	0.005 ppm	16 h	Fluorescence Plate Reader
Hurdebise et al. [28]	zntA-gfp	660 ppb	3 h	Flow cytometer
Kang et al. [29]	zntA-gfp	112.4 ppb	2 h	Fluorescence microscopy
